# The Roberts-Hawley Goat Lymph Compound

**Published:** 1901-03

**Authors:** Willis P. King

**Affiliations:** President American Animal Theraphy Association, San Antonio, Texas


THE
### TEXAS MEDICAL JOURNAL.

ESTABLISHED JULY, 1885.

PUBLISHED MONTHLY.—SUBSCRIPTION $1.00 A YEAR.

VOL. XVI. AUSTIN, MARCH, 1901. No. 9.

### Original Contributions.

For Texas Medical Journal.

### THE ROBERTS-HAWLEY GOAT LYMPH COMPOUND.

**What it is; How it acts; Its limitations; Precautions in its use.**

BY WILLIS P. KING, M. D., PRESIDENT AMERICAN ANIMAL THERAPHY ASSOCIATION, SAN ANTONIO, TEXAS.

Having recently given the profession of Texas a glimpse of what this remarkable remedy is, and as to what it does, I feel that every inquiring practitioner would like to know something more about it.

WHAT IT IS: It is the lymph fluid from the lymphatic glands of the young, healthy, male goat, taken from the animal, as far as possible, under chloroform, after the goat has been fed for some months on such foods as tend to increase the size, contents, purity and richness of the fluid—combined with cells from the medulla oblongata, spinal cord and gray matter, thyroid, supra-renal capsule extract and spleen pulp of the goat, obtained by maceration and compression; the contents of the thoracic duct and *receptaculum chyli,* and five per cent. of bull semen, all combined in certain proportions and put up in blood serum and carbonized water; and "fixed," or put in a state in which, after being hermetically sealed, it will keep indefinitely without spoiling or degenerating.

Having been practically resurrected myself, by using this lymph treatment, after a long, debilitating and most serious sickness of six years—first, a terrible breakdown from brain and nerve exhaustion; and, afterwards, almost destroyed by syphilis contracted in a surgical operation—and made well and strong again, and from a broken-down, prematurely old man, made to feel young and vigorous again, I determined to use it in other appropriate cases and, if possible, devote my life to giving it to the hopeless, helpless and heretofore incurable cases, and to the work of bringing it to the notice of physicians everywhere that the influence of my voice and pen may reach.

Laying aside the question of financial gain I do not believe that any good man can devote himself to any work in life in which so much real, lasting good may be done to others.

I wish to say here, in the outset, that I have not been uniformly successful. There are cases in all the classes of cases to which this remedy is applicable, which are so near death's door, in which so many destructive pathological changes have occurred, that to cure them is impossible. This may be said of this remedy which may not be truthfully said of any other remedy that I know of; it does good, in some way, in almost every case in which it is given. The people who suffer from diseases of nutrition, which, as the name would indicate, are wasting diseases, and those who suffer from diseases due to degenerative changes in the brain and nervous system are nearly all great sufferers. Diseases which waste the body produce a more or less profound impression upon the nervous system. Nerve starvation is the one almost constant event that occurs at some period in such diseases; and a starving nerve does just what a starving child does, it cries; and every pain is the cry of a nerve. The degenerative changes that come with old age are often the cause of pain in some part of the body and many old people suffer daily, often with the conviction that their suffering is a necessary incident to their growing old, and that there is no help and no remedy for it. To such this lymph compound is simply a priceless boon, for it relieves such suffering in all the classes I have mentioned—not as opiates relieve them, but in a perfectly natural way—by supplying vitalized nutrition to the crying nerve, to the spinal cord, or to the starved area in the brain. In such cases the action of this remedy is most beautiful, and the most gratifying results are obtained in its exhibition of any remedy I ever administered.

Now I shall give, in as brief a way as possible, some of the results I have obtained:

*Chronic Interstitial Nephritis.—Case 1:* H. J., cigar maker, age 31. Had not worked for fourteen months. Legs and face enormously œdematous. Left ventricle of heart dilated, dyspnœa, unable to sleep in a recumbent posture, weak, neurasthenic, despondent, hopeless. Urine loaded with albumen and tube casts. Treatment thirty days, seven to eight drops twice a day, adjuvant by mouth. Under protest of my partner, he went to work on 8th day. Albumen and tube casts disappeared from urine in middle of third week. At end of thirty days, to all appearances, the patient was perfectly well. Compensation of heart perfect; he sleeps nine hours without waking. By advice he took another half treatment as a matter of precaution.

*Case 2.* J. P. H., age 48, conductor. *Chronic interstitial nephritis,* paralysis of anterior muscles of legs, two years, and of left side of face, one year. Usual findings of albumen and tube casts in urine. Had been treated by a specialist in Saint Louis with static electricity, strychnia and massage for six months, without results.

Treatment: Eight drops twice a day, with adjuvant by mouth. Paralysis of legs relieved on fifth morning and of face on tenth morning. On fourteenth day he received a letter from the chairman of a State political committee, asking him to come to Saint Louis—he being a candidate of the dominant party for a State office. He overruled my objections and went—taking with him a week's treatment, and a letter to his Saint Louis physician. He refused to return for further treatment, saying, in a letter: "I am as well as I ever was in my life." I knew better; for, while the action of the remedy in his case had been prompt, and remarkable beyond anything I had ever seen, I knew that it was inconceivable that a cure should have been effected in so short a time. In November, 1900, after he had been elected, he went down in Saint Louis with uremic coma, and died—another unfortunate instance in which the patient thought he knew better than his doctor.

*Case 3.*—P. W., age 34, lawyer. *Multiple neuritis* (alcoholic). Complications, acute congestion of kidneys and acute hypertrophy of liver. Came to me about August 1, 1900, after having been in a local hospital at his home for one month.

He could scarcely walk; extreme pain and anæsthesia in lower extremities. I put him in charge of a neurologist of ability who treated him one month with static electricity, strychnia (hypodermatically) and arsenic by mouth. On September 1st, when I returned from American Animal Theraphy Association meeting at Chicago, I found him suffering from illusions, hallucinations and delusions—*acute hallucinatory mania,* and taking three grains morphine by hypodermic injection every twenty-four hours. I again took charge of the case and gave him first dose of Lymph (ten drops in the calf of left leg) on evening of September 2nd. Within a few days I was giving him sixteen drops morning and evening and ten at noon. This large dose kept up only a few days. I began at once gradually to withdraw the morphine, and to substitute water, with an occasional dose of codeine phosphate, when we knew he was suffering greatly. He improved from the first, and within two weeks his mind was perfectly clear. On September 13th I gave him the last (1-16 gr.) dose of morphine, and on October 13th, one month later, informed him of the trick we had played on him. Treatment was continued about two months, when he returned to the practice of his profession, a perfectly well man. Without the use of this remedy I should have expected to have to deal with a case of *cirrhosis of the liver* and a *chronic interstitial nephritis.* As it was, both of these organs resumed their normal size and functions.

*Case 4.—Fatty Infiltration of Heart—Arterio-Schlerosis:* W. S., age 46. Has been a hard drinker. Has suffered for more than a year from attacks of *angina pectoris.* Apex beat of heart far to left of nipple, heart sounds characteristic, the radial artery could be plainly felt after blood column was shut off.

Treatment: Eight drops twice a day, with adjuvant by mouth for two weeks, and ten to twelve drops, once a day, for two weeks. Never had a serious attack of *angina* after beginning treatment and had no attack after four or five days. Improved in strength, slept better, and was better in every way. Patient was not able to (or would not) pay for remedy, and I had to stop giving it. He is still feeling well (after two months), but will relapse in time, as he should have had the treatment for at least ninety days.

*Case 5.—Melancholia:* Mrs. T., age 34, mother of nine children in fourteen years—seven living, last a babe seven months old. Had been suffering from melancholia for several months. Had a number of physicians, all of whom advised that she be taken to an asylum. She was fat, pale, flabby, constipated, with weak and rapid heart. Melancholia due to general anemia and overwork for her family. Treated by Dr. C. M. Decker of San Antonio. Eight drops twice a day with adjuvant by mouth. Recovered mind during night between fourth and fifth day. While she would not notice her children, not even her sick baby, she was afraid of her family, wanted to go away, and was up, trying to dress herself at all hours of the night, she was weeping over her sick baby on the fifth day, and, in spite of Dr. Decker's orders, went to the wash tub on the seventh day, and to the sewing machine on the eighth,—trying to do some thing for her long neglected household. Took ten day's treatment, (four drachms of Lymph), and her husband refused to pay for more, saying, "Vat I vant mit more of dot costhly medicine? My vife, she vell." She is still well now, three months later; but I fear a relapse, as she should have been treated at least sixty days.

*Case 6.—Cystic Degeneration of Ovaries:* Miss B. M., age 27. I removed a cystic left ovary from this young lady one year ago and fastened stump of broad ligament and tube in abdominal wound, in order to retain in position a long retroflexed uterus. The cystic ovary had been crowded down underneath the uterus. The other ovary was healthy at that time. Eight months later trouble began in right ovary. When she came here from her home (1000 miles) she had been confined to her bed nearly all the time and had not worn a dress, except a simple loose wrapper, for three months. Examination revealed uterus still in good position, but right ovary evidently undergoing cystic degenerative process. The whole pelvic region was extremely tender to touch, though there was no thickening, nor any other evidences of any septic inflammatory process. In fact the pathological condition was not inflammatory, but degenerative, and the extreme tenderness was due to nerve exhaustion and irritation,—*neuritis.* The cystic degenerative process was due, no doubt, to the condition of the local nerve supply, and this local nerve supply had been injured by the long retroflexed uterus and the daily reflex "nagging" of this malposition, its interference with the local blood supply, etc. She was prepared and put upon treatment—eight drops twice daily—about two weeks ago. On the fifth day she put on her dress; on the eighth day she walked three and one-half miles, and on the ninth day nearly twice as far (both estimated), with a friend in seeing the sights of San Antonio. All pain and tenderness is relieved, and she is eating, sleeping, walking and going where she pleases. When she came to me her face was mottled and splotched. In a week or ten days her complexion cleared up, that care worn expression left her, and her skin is now as clear as a baby's. This is a typical case of the kind in which the patient goes to bed and remains there, in a condition of chronic invalidism, for years and years. I feel sure that I shall send this young woman home perfectly well and happy.

*Case 7.—Tuberculosis of Lungs and Tubercular Laryngitis:*

L. C., age 31, machinist. This young man had been away from his home for three years. Examination reveals tubercular infiltration of apices of both lungs, the left being the worst. Husky, half-whispering voice indicates *tubercular laryngitis.* General emaciation and physical weakness, face pale. Sputum has been examined by a competent pathologist and *tubercle baccilli* found.

Treatment: Six drops twice a day with adjuvant by mouth. Remedy carried to seven and eight drops within a week, when patient showed great physical weakness with headache and insomnia. This marks his as a case of idiosyncrasy, or unusual susceptibility to the influence of the remedy.

Twice, within three weeks, I have been compelled to stop the treatment for a few days and, finally, I have settled down to four or five drops once a day. His lungs have cleared up so that there is scarcely any evidence of infiltration, his voice is normal, resonant and clear, his eyes bright, there is a red healthy flush on his face, he sleeps all night without coughing, and he shows every evidence of a man who is practically well; and yet he is weak, (from the remedy, no doubt), and I can put him flat of his back with two doses of eight drops. This is a most singular condition and one I can not explain.

*Case 8.—Chronic Tuberculosis:* Miss A. L., age 23. Has suffered from pulmonary tuberculosis for many years; was treated in Saint Louis with some of the serums or anti-toxins. Came here one year ago. She was weak, coughed a great deal, suffered from a severe naso-pharyngeal catarrh, was hysterical and often wept all night; her face was pale, her hands and feet cold, and her condition generally was bad. Evidences of infiltration at apices of both lungs, though the tubercular condition was not progressive, but seemed to be at a standstill.

Began treatment three weeks ago. Her improvement was marked and rapid from the first. On about the eighth day she simply cleared out the nose and naso-pharynx,—blowing out large quantities of muco-pus; and, after that, all discharge from this source ceased. She regained her strength, began to sleep well, her hysterical symptoms disappeared, cough ceased, face began to show the healthy pink and red, and I regard the young woman as being well.

Another case very much like hers, (except the hysterical symptoms),—her roommate; has about the same history as to general and local conditions; she has improved quite as rapidly as the other.

*Case 9.—Spasmodic Asthma, Eight Years; Neurasthenia, Three*

*Years:* R. G. A., age 48, physician. This man has been away from home a hopeless, helpless, suffering wanderer, for three years. When he came to me he was as complete a picture of despair as I ever saw. He was taking two grains of morphine every twenty-four hours. He had to. There was no relief from the terrible spasms of smothering and coughing except morphine. His face was mottled and spoltched, showing a weak and poor circulation, his extremities were cold and his sleep was poor.

Treatment: Eight drops twice a day with adjuvant, teaspoonful after meals, by mouth. On the third day he did something that no person suffering from general neurasthenia can do without this remedy,—he laid down and slept one hour in the forenoon and two hours in the afternoon. He dropped off one and one-half grains of his morphine within the first four days, and the other half grain within ten days. At about the end of the second week he ceased to have the severe attacks of asthma at two a. m., as formerly, and has not since had any spasmodic attacks at all. He showed, for a time, some evidences of the thickening of the walls of the terminal bronchioles,—due to a secondary catarrhal bronchitis; but that has about disappeared, and, within a short time, this once suffering man will return to his home and resume his business.

*Case 10.—Hemiphlegia:* G. S. H., lawyer, age 50. This man, (who is the law partner of Ex-Governor William J. Stone, of Missouri, and has been for twenty years), suffered from neurasthenia for about twelve years. His business was unusually arduous; and, two years ago, he suffered a stroke of left side paralysis. After I became well, I wrote him and induced him to take this treatment. Before treatment his condition was as follows: He still dragged the left foot, and could not shut the left hand; suffered from marked despondency, insomnia, and great physical weakness After the usual two days preparation he began the treatment,—taking eight drops twice a day, with adjuvant as usual. He recovered the use of his hand on the fifth morning, and ceased to drag his foot on the eleventh morning. He very soon recovered from his despondency and insomnia, and is today perfectly well,—sleeps well, eats what he wants, and is engaged in a large law practice to which he devotes his time, and is as well as ever in his life.

I have had a number of cases of failure in different classes, most of them due to the bad weather we have had,—for I find that, as a rule, this remedy does not demonstrate its therapeutic worth when the skin is not acting. I was at a loss for some time to understand why it was that, whenever there was a "norther" here, all of my patients came in on the second morning afterwards not doing so well. One did not sleep well the night before, one had a headache, another was feeling weak, and in four cases out of five there was something wrong. Having watched my cases and studied the conditions under such circumstances a number of times, I have come to the conclusion that when the weather becomes cold and the functions of the skin are suppressed, it is best to withdraw the remedy until the weather becomes settled and warm again.

HOW IT ACTS: The Lymph is a capillary dilator and a tonic to the fixed tissue cells. Even in an old man the palms, fingers and nails will become pink within a week or ten days after taking the Lymph. This same condition occurs, no doubt, in the capillaries of the brain and spinal cord and other organs. In cases two and ten (referred to above), there can be no question as to what the action of the remedy was. The capillary arteries in the motor area of the brain had been closed by atheromatous degeneration; nutrition had been cut off from certain centers and those centers had ceased to act; and, of course, the parts controlled by those centers became paralyzed. When the remedy, by its action as a capillary dilator, opened up those small vessels and nutrition was carried to the center, this center, being nourished, resumed its normal function. In case second, the left side of whose face had been paralyzed for one year, it was the origin of the seventh nerve (the motor nerve of the face), that was involved, and when, during the ninth night, nutrition was carried to the origin of this nerve, the paralyzed face at once became straight. It was as bad a case of facial paralysis as I ever saw,—the face being the flattest and showing the least mobility, and the eye red and staring; and yet, on the next morning, no one could tell that there had ever been anything wrong with his face.

There is no doubt in my mind that the Lymph cells are charged with vitalized nutrition—the very highest form of nutrition that the body uses. This *vitalized* nutrition, no doubt, nourishes the brain and nervous system. In many chronic, debilitating and wasting diseases this form of nutrition seems to be lost, or rather, the sick one does not elaborate it any more. In such cases we find that pain is an important symptom in the disease—periodical headache and pains in the back, hips, thighs, legs and feet. I find this condition in many of the cases of chronic tuberculosis I meet with here. In every case the Lymph has relieved those pains within five to ten days. It gives relief, no doubt, by nourishing the brain, spinal cord and the nerves themselves. This is my theory, and if it be wrong, no one is responsible for it but me.

CAUTION: There are cases that seem to be unusually susceptible to this remedy; and, if the doctor is not warned, he will become discouraged when he meets with such a case. In such cases and after the remedy has been given from three days to one week, some one, or maybe all of the following symptoms will appear: the patient will become weak, will suffer from headache, insomnia, and sometimes from a vague, undefined, general distress. Under such circumstances the remedy should be withdrawn, the bowels purged out, baths taken, the body massaged and then go back to two or three drop doses, twice a day, or to one dose of four or five drops each day.

Physicians should insist on the gymnastic exercises of the body—the flexing and extending of the arms and legs, stooping and raising the body; and, when there is some one in the family who will do it, the body of the patient should be kneaded and rubbed all over at bedtime. This is best after a warm bath. Light suppers should always be taken.

Sometimes patients will cease to be hungry after taking the remedy a week or ten days. No uneasiness should be felt when this occurs, as the Lymph is feeding the tissues, and they, being satisfied, do not care for other food.

If a doctor were to ask me: "To what class or classes of cases is this Lymph applicable as a remedy?" I would answer: "To all diseases of nutrition, such as tuberculosis, chronic rheumatism, diabetes, "Bright's" disease, and to all chronic wasting diseases of the brain and nervous system—such as paralysis, paresis, locomotor ataxia, epilepsia, neurasthenia, and to all cases of interstitial hyperplasias, the result of long continued inflammatory diseases, to all connective tissue overgrowths, and to all chronic diseases of the stomach and alimentary canal.

The one mistake that almost every doctor makes in beginning the use of this remedy, is in running the dose up too rapidly, and therefore, giving too much of it; and the second, and worst mistake that both patient and doctor make, is in expecting too much, and to expect such a result as a complete cure in too short a time. While the remedy acts quickly and remarkably in some cases, it does not do it in all cases, and the doctor should prepare his patient's mind to be patient and not to expect to be cured of an old chronic disease of many years standing in a short time.

Again the patient's mistake is in expecting to be cured too cheap.

I have had patients to boast to me that they had paid out thousands of dollars to arrant quacks and humbugs; and, when told that this treatment was costly, and that they would have to pay $60.00, $75.00, and sometimes, when their financial circumstances would warrant it, $150.00, for a five or six weeks treatment, they would seem to lose their breath and almost have a fit. The remedy itself, as compared with others, is costly; but, when we consider what it will do in an appropriate case, it is cheap. I would have considered it cheap at one dollar per drop, could I have known beforehand that it would do for me what it did.

ADDENDA: Finding, upon calculation, that I had some space of that assigned me by the editor still unused, I desire to add a number of things that I think are important.

1. The "adjuvant" mentioned in this paper comes with the Lymph. It is composed of a number of animal products, and is put up in a compound tincture of the bitter barks. It is taken by the mouth, a teaspoonful three or four times a day, while the Lymph is being given hypodermatically.

2. I find that many persons who suffer from chronic diseases seem to be filled with toxins, the result of accumulated waste products due to the disease, and which the emunctories, in the weakened condition of the system, have failed to throw off. If the Lymph is pushed to large doses early in the treatment these toxins often seem to be set free by the action of the Lymph as a tonic to the fixed tissue cells, and it will sometimes produce fever, and sometimes a general distress. In such cases it is important to remember that elimination must keep pace with the progress of the remedy; hence the remedy must be withdrawn, the bowels purged out, baths administered, massage used and gymnastic exercises taken. Then begin, after two days with a very small dose, two to four drops, twice a day, and gradually raise the dose again.

In one case where the person had rheumatism years ago, and whose disease was not well defined (he felt bad, he was stiff, had headache, ached all over, couldn't sleep, etc.), I am sure I set up an acute rheumatism by using too large a dose, thereby dissolving the uric acid crystals and other poisonous causes of rheumatism, and precipitated it into his circulation. Here a great deal more good would have been done by proceeding slowly; but, people who come for the treatment seem to be always in a hurry, and they want the mischief of a decade of chronic diseased conditions undone in a week.

3. I have not spoken as I feel that I should do regarding the value of this remedy in old age and in the ailments and disabilities incident thereto. It seems to possess the power to almost make the old man young. Old people suffer from many ailments that are the result of the long wear of the system, and of the nervous system especially; for instance, the backaches in both sexes, and the bladder irritation and frequent mioturition of old men. These conditions, I find, are due solely to local neurasthenias. That part of the nervous system which supplies the genito-urinary apparatus, the lumbar region, and, sometimes, one side of the scalp, becomes worn out and poorly nourished, and a pain or an ache is the result. It has gotten to be an almost settled conviction in the medical mind, that as these conditions are due to old age, nothing can be done to permanently relieve them. The Lymph treatment has relieved every case in which I have tried it. I have tried it in a number of cases of hemicrania, with practically perfect results. Such cases of chronic headaches (generally in old ladies), are due, as a rule, to exhaustion of a single nerve that supplies the scalp, and may and can be relieved and cured promptly.

Again, in the so-called "chronic inflammation of the bladder in the female" I find that bugbear which no physician wants to treat. I find that ninety per cent. are due to local neurasthenia alone. Many poor, suffering women have been bedridden for years with this terrible malady, and many become morphine habitues, when relief may be obtained by using this remedy ten days, and a permanent cure effected by its use thirty or forty days. In the absence of this remedy strychnia alone—given in large doses, hypodermatically, (the 1-24 three times a day), will do more good than any of the other old time remedies that I have ever used. I recommend the strychnia in such cases only as cannot for any reason take the Lymph. The Lymph treatment is far better than anything else—the strychnia ranks second with me.

